# A thermogenic botanical composition containing *Citrus aurantifolia* fruit rind and *Theobroma cacao* seed extracts improves body composition in overweight adults: a clinical investigation

**DOI:** 10.29219/fnr.v69.12159

**Published:** 2025-06-24

**Authors:** Amulya Chadalavada, Yean Kyoung Koo, SukJin Kim, Sudipta Veeramachaneni, Guru Ramanathan, Amulya Yalamanchi

**Affiliations:** 1 Department of General Medicine, Aditya Multi Speciality Hospital, Guntur, India; 2 Department of R&I Center, COSMAXBIO, Seongnam, Republic of Korea; 3 SV Scientific, One PPG Place, Suite 3300, Pittsburgh, PA, USA; 4 Pharmacology-Based Clinical Trials Laboratory, Pennington Biomedical Research Center, Baton Rouge, LA, USA; 5 Department of General Medicine, Yalamanchi Hospitals and Research Centre, Vijayawada, India

**Keywords:** body composition, glucagon-like peptide-1, resting metabolic rate, Theolim™, thermogenic botanical combination

## Abstract

**Background and objective:**

CL19183, or Theolim™, is a novel, proprietary combination of standardized extracts of *Citrus aurantifolia* fruit rind and *Theobroma cacao* seeds. Earlier, CL19183 supplementation demonstrated thermogenic activity and weight loss in high-fat diet-induced obese rats. This randomized, double-blind, placebo-controlled, multicenter clinical study (RCT) assessed whether CL19183 supplementation reduced body weight (BW) and improved body composition (BC) in overweight adults.

**Methods:**

The present study recruited 120 overweight male and female subjects (25–55 years) [body mass index (BMI) of 25–29.9 kg/m^2^] and randomly assigned to receive daily either CL19183 (450 mg; *n* = 60) or a matched placebo (*n* = 60) over 16 weeks. The primary efficacy outcome measure was BW reduction in the intention-to-treat (ITT) population. Other efficacy measures included BC using Dual-energy X-ray absorptiometry (DEXA), waist and hip circumferences, resting metabolic rate (RMR) using indirect calorimetry, serum lipid profile, and serum biomarkers utilizing enzyme-linked immunosorbent assay (ELISA). The safety parameters were performed, including complete serum biochemistry, hematology, and urine analysis.

**Results:**

Post-trial, CL19183 supplementation resulted in significant reductions in BW (4.25 ± 1.35 vs. 0.96 ± 1.18 kg; *p* = 0.0001; CI [confidence interval]: 1.47, 8.59) and BMI (1.57 ± 0.53 vs 0.36 ± 0.46 kg/m^2^, *p* < 0.0001; CI: 0.87, 2.11), from baseline as compared to placebo. Similarly, total body fat (4.28 ± 1.56 vs. 0.85 ± 1.06 kg; *p* < 0.0001; CI: 2.35, 7.79) and fat percentage (*p* < 0.0001) were also reduced from baseline in the CL19183 group vs. placebo. At baseline, after a single dose of CL19183 administration and after 16 weeks, RMR was significantly increased (*p* < 0.0001 vs. placebo). After 8 and 16 weeks of supplementation, CL19183 significantly increased serum adiponectin and glucagon-like peptide-1 and decreased ghrelin levels vs. baseline and placebo. No major adverse events were reported.

**Conclusion:**

CL19183 supplementation was well-tolerated and led to significant BW reduction and improvements in BC over 16 weeks.

## Popular scientific summary

- CL19183, or Theolim™, is a thermogenic botanical ingredient, a combined extract of *Citrus aurantifolia* fruit rind and *Theobroma cacao* seeds.
- Sixteen weeks of CL19183 supplementation reduced body weight and improved body composition and anthropometric parameters in overweight adults.
- CL19183 improved insulin sensitivity, serum lipid profile, and adiponectin, GLP-1, and ghrelin levels.
- Long-term CL19183 supplementation is safe.

The projected prevalence of obesity worldwide would be 18% in men and 21% in women by 2025 (1). Excess body fat is widely associated with non-communicable diseases, including hypertension, insulin resistance, cardiovascular diseases, hyperlipidemia, and non-alcoholic fatty liver conditions (2, 3). An increase in body fat mass is generally attributed to an imbalance between dietary calorie intake and energy expenditure (4). Generally, the methods for controlling excess fat mass include exercise, lifestyle modification, and pharmacological and surgical interventions. While these measures can be effective, they are often expensive, time-consuming, and associated with side effects. In particular, the pharmacological interventions lead to side effects such as steatorrhea, fecal urgency, insomnia, paresthesia, headache, nausea, dizziness, pancreatitis, dyspepsia, dry mouth, increased blood pressure, and heart rate (5, 6).

Natural products are generally considered as an alternative to pharmacological drugs with minimal or no side effects (7, 8). Herbal medicines are reported to reduce body weight (BW) through multiple mechanisms, including appetite suppression and reduced energy intake, enhanced thermogenesis and resting energy expenditure (REE), inhibiting pancreatic lipase and reducing fat absorption, increasing lipolysis, and decreasing lipogenesis (9, 10).

Recently, Kundimi et al. investigated the effects of CL19183 (also referred to as LN19183 or Theolim^TM^), a proprietary, synergistic composition consisting of aqueous-ethanol extracts of *Citrus aurantifolia* fruit rind and *Theobroma cacao* seeds. It increased thermogenesis by enhancing Fibroblast growth factor 21 (FGF-21) production in adipocytes and by inducing uncoupling protein-1 (UCP-1) and peroxisome proliferator-activated receptor-gamma coactivator-1 alpha (PGC-1α) (11). Subsequently, Ammatalli et al. demonstrated that CL19183 supplementation reduced the BW of obese rats on a high-fat diet (HFD) by increasing REE. Also, in a randomized, double-blind cross-over clinical study, an acute CL19183 supplementation increased metabolic rates at rest and with exercise in overweight male and female subjects (12).

Citrus fruit rind or peel contains flavonoids, pectin, and Vitamin C. *Citrus aurantifolia* fruit rind flavonoids, including hesperidin, have been reported to exert anti-obesity efficacy by reducing appetite and improving lipid metabolism (13–15). The consumption of citrus and its extracts has been reported to reduce body mass index (BMI), waist and hip circumference, and BW, with more pronounced effects at higher doses (16, 17). Similarly, cocoa has been shown to prevent HFD-induced obesity by modulating lipid metabolism – specifically, by decreasing fatty acid synthesis and transport while enhancing thermogenesis in the liver and white adipose tissue.

The present clinical investigation aimed to determine the long-term efficacy of CL19183 on reducing BW and BMI, improving body composition (BC) including body fat, lean body mass (LBM), waist and hip circumferences, and increasing resting metabolic rate (RMR) in healthy overweight volunteers. Moreover, this study also evaluated the long-term safety of CL19183 supplementation by measuring safety parameters, including serum biochemistry, hematology, and urinalysis.

## Materials and methods

### A proprietary phytoceutical composition, CL19183

CL19183, a proprietary combination of *Citrus aurantifolia* fruit rind and *Theobroma cacao* seed extracts, was manufactured at a good manufacturing practice (cGMP)-certified facility of Laila Nutraceuticals, Vijayawada, India. Taxonomically authenticated voucher specimens of *C. aurantifolia* fruit rind (LNR6824) and *T. cacao* seeds (LNR6924) are archived in the Taxonomy Department, Laila Nutraceuticals (Vijayawada, India). The extracts of *C. aurantifolia* fruit rind (CA) and *T. cacao* seed (TC) were blended at a 2:1 ratio. CL19183 contains 80% herbal blend and 20% excipients (18% glucidex and 2% syloid).

### Ethical conduct

**Fig. 1:**
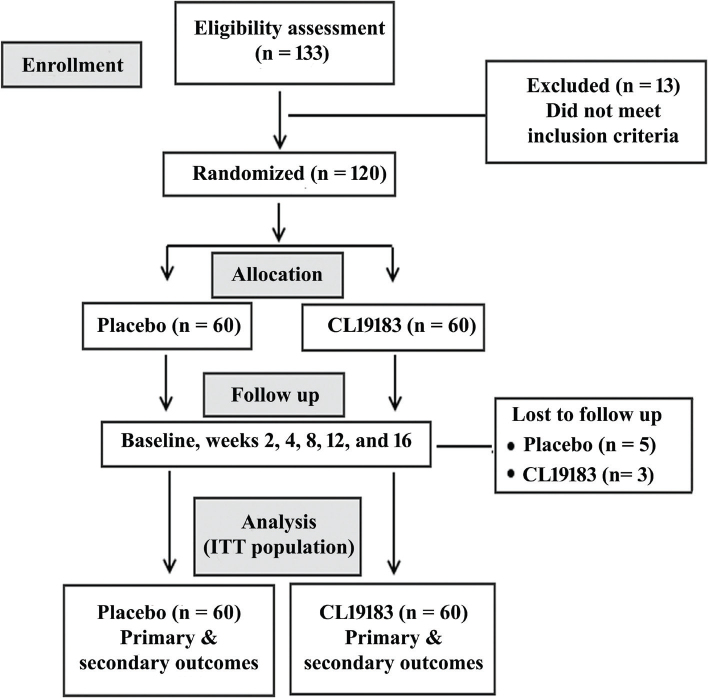
A CONSORT flow diagram presents the sequential steps of the 16-week trial. The efficacy analyses were performed with the intention-to-treat (ITT) population.

This randomized, double-blinded, placebo-controlled, multicenter clinical trial was registered with the Clinical Trial Registry of India (Registration No: CTRI/2023/06/054552; June 30, 2023). This investigation was conducted in three hospitals in Guntur, Andhra Pradesh, India, and the study protocol was approved by the institutional ethics committees of Aditya Multispeciality Hospital (ECR/1347/Inst/AP/2020; on June 19, 2023), Samishta Hospitals & Research Institute (ECR/1497/Inst/AP/2021 on June 19, 2023), and NUHA Hospitals (ECR/1593/Inst/AP/2021 on August 14, 2023). This study followed the principles of the Declaration of Helsinki, International Council on Harmonization (ICH)-Good Clinical Practice (GCP) guidelines. The study flow followed the recommendations of Consolidated Standards of Reporting Trials (CONSORT) (Fig. 1).

### Participant enrollment, consent, randomization, and blinding

Participants visiting the outpatient departments of the clinics were selected for screening after appropriate due diligence. Male and female subjects voluntarily signed the informed consent forms and were screened for eligibility for this study based on inclusion and exclusion criteria (Supplementary Table S1). Briefly, the criteria for eligibility of the healthy subjects were determined based on their medical history and routine laboratory examinations. The subjects had a sedentary lifestyle, no regular athletic or sports activities, and were willing to participate in walking (5 days a week, 30 min per day) during this investigation. This study did not enroll any subjects who participated in weight loss programs or used weight reduction or thermogenic supplements. Pregnant women or lactating mothers did not participate in this study.

The enrolled participants (*n* = 120) were equally allocated to either placebo or CL19183 groups using permuted block-randomization code generated by the PROC-PLAN procedure in the Statistical Analysis System (SAS) program. An independent pharmacist controlled the randomization details. The investigators, study monitors, and the study participants were blinded to the randomization and treatments. Randomization codes were broken only after locking the data following the completion of the study.

### Sample size and power calculation

Sixty subjects per group (*n* = 60) were chosen for this study, which satisfied the 90% power criteria at the 95% confidence interval (CI) with an anticipated dropout rate of 15%. The mean BW change (d) of 2.0 kg and the pooled standard deviation (SD) of 3.0, based on an earlier multicenter clinical study (RCT) in an overweight population (18), were used for the power calculation of the present study.

### Placebo or CL19183 supplementation

CL19183 or matched placebo capsules (hard gelatin, 0-size) of identical sizes, weights, and colors packed in coded white high-density polypropylene (HDPE) bottles. Recruited subjects were advised to consume either one placebo or CL19183 capsule (450 mg/day) after breakfast over 16 weeks. The subjects took the placebo or CL19183 capsules on the evaluation days before breakfast. Each placebo capsule contained (w/w) brown dextrin (50%) and maltodextrin (50%).

### Follow-up visits

After screening (visit 1), randomization and baseline evaluations were conducted (visit 2). Following visit 2 (baseline), this study had five follow-up evaluations on week 2 (visit 3), week 4 (visit 4), week 8 (visit 5), week 12 (visit 6), and week 16 (visit 7).

### Compliance

The placebo or CL19183 capsules were stored at room temperature in a dry, cool, and dark place. The project coordinators distributed the placebo and treatment capsules to the recruited volunteers at baseline and on visits 3, 4, 5, and 6 of the study. They maintained the data entry and were endorsed regularly by the principal investigator (PI). The PI regularly signed the accountability log. Study participants were instructed to maintain their routine regular diets and refrain from consuming supplements, food, or beverages that may enhance weight loss. All subjects regularly maintained individual daily diaries and recorded details of food and capsule intakes, daily activities, and any adverse events. Dietary carbohydrate, protein, fat, and total calorie consumption were calculated and recorded using DietSoft, a dietary calculation software (Invincible Ideas, Noida, Uttar Pradesh, India).

The project coordinators and PI reinforced compliance at every study visit to ensure the maximum possible adherence to the study protocol. The participants returned all unused capsules at each follow-up visit, and their attendance at each visit was recorded to ensure the participants’ IP-related and participant compliance with the study protocol.

The PI determined the physical health of all study participants by checking the physical signs of any adverse drug reaction. At baseline and each follow-up visit, safety was ascertained by medical check-ups.

### Data management and monitoring

The participating site was regularly monitored by well-trained clinical research associates in compliance with GCPs. Data verification during on-site monitoring visits, data entry, and validation (e.g. range checks) was performed to ensure the study compliance. All documents and data collected from participants were kept confidential in a separate and secure location.

### Subject withdrawal criteria

Participants could withdraw at any time if they wished to discontinue their participation. The withdrawal of subjects from the study was considered if the subject had withdrawn consent or the investigator considered withdrawal in case of non-compliance with IP protocol violation or loss to follow-up.

### Efficacy analysis

#### BW and BC

The participants’ BW was measured on all the visits using a digital weighing machine (Model No. HN-289 Omron, Mumbai, India).

The Dual-energy X-ray absorptiometry (DEXA) (Model: Lunar DPX NT, GE Healthcare, Chicago, IL) was used to assess BMI, total fat mass, total body fat percentage, and LBM, following the standard protocol (19).

#### Waist and hip circumferences

Following the World Health Organization guidelines, waist circumference (WC) was measured at the midpoint between the lower margin of the last palpable rib in the midaxillary line and the top of the iliac crest. Hip circumference was measured at the largest circumference of the buttocks (20).

#### Resting metabolic rate

Resting metabolic rate (RMR) was measured using a non-re-breathable silicon mask attached to a human metabolic cart (Iworx Systems Inc, Dover, NH) and expressed in kilocalories per day [kcal/day]. All tests were performed according to current methodological guidelines available (21). Briefly, participants stayed motionless (supine position) on a bed for 15–20 min before the first measurement (acclimation period before the RMR assessment). The participants confirmed that they did not perform any moderate and/or vigorous physical activity in the past 24–48 h. Participants were instructed to breathe normally and not to talk or fidget during the assessments. The room temperature and relative humidity were maintained at 25°C and 38%, respectively, and similar test conditions were strictly followed for every participant and during all visits. The device determined the RER by capturing the volumes of CO_2_ exhaled and O_2_ utilized per minute. The RER was computed as VCO_2_/VO_2_ according to Weir’s equation (22).

#### Homeostatic model assessment for insulin resistance

The participants’ homeostatic model assessment for insulin resistance (HOMA-IR) was calculated by fasting glucose and insulin levels using the following formula (23):

HOMA-IR = [glucose (mg/dL) × serum insulin (mUI/L)/405]

#### The profile of mood states-short form

This 37-item questionnaire is a validated and widely used tool to assess psychological discomfort and emotional states. Each item of this self-report questionnaire was scored on a five-point Likert scale (0–4) – 0 = not at all, 4 = extremely – based on the feelings/mood states that participants had felt in the past 24 h (24).

The DEXA scan, RMR, Respiratory Exchange Ratio (RER), and HOMA-IR were measured at baseline, week 8, and week 16. Waist and hip circumferences and the The Profile of Mood States-Short Form (POMS-SF) questionnaire were assessed at baseline and all follow-up visits.

#### Serum biomarkers

Serum adiponectin, ghrelin, and glucagon-like peptide (GLP-1) levels were determined by specific and sensitive EIA methods using human ELISA kits. Adiponectin assay kit (Cat#DRP300) was procured from R&D Systems, Minneapolis, MN, and ghrelin (Cat# E-EL-H1919) and GLP-1 (Cat# E-EL-H6025) assay kits were procured from Elabscience, Houston, TX. The immunoassays were performed following the protocols provided by the manufacturers. The assay sensitivities of the adiponectin, ghrelin, and GLP-1 kits were 0.891 ng/mL, 0.1 ng/mL, and 0.94 pg/mL, respectively.

For lipid profiling (total cholesterol, triglyceride, high-density lipoprotein [HDL] cholesterol, very low-density lipoproteins [LDL], and LDL cholesterol), serum samples were evaluated. Serum lipids and protein biomarkers were measured at baseline and on the 8th and 16th weeks of the study.

### Safety assessments

For the assessment of CL19183 safety, a series of parameters were evaluated in serum and whole blood of all participants at the baseline and the end of the study. Biochemical and hematological parameters were measured using the automated analyzer COBAS INTEGRA (400 PLUS) auto clinical chemistry analyzer, Roche Diagnostics Ltd. (Rotkreuz, Switzerland) and the hematological counter MINDRAY (BC-5380) auto hematology analyzer (Shenzhen, China), respectively. Urine analysis was carried out using UroColor Strips (Standard Diagnostics, Kyonggi-do, Korea) and by microscopy of sediment. Vital signs (blood pressure, oral temperature, pulse rate, and respiratory rates after sitting for 5 min) were measured at all study visits.

### Statistical analysis

All randomized participants who received at least one dose of CL19183 or a placebo capsule were included in the intention-to-treat (ITT) analysis for efficacy and safety measures using SAS 9.4 (SAS Institute, NC, USA). The last observation carried forward (LOCF) or baseline observation carried forward (BOCF) methods were used to address missing values (25). The descriptive statistics were performed, and the data are expressed as the mean ± SD. The normality of the variables was confirmed using the Shapiro–Wilk test. Baseline characteristics were compared using the unpaired-t test. For the efficacy and safety analysis, the ‘intergroup’ and ‘intragroup’ comparisons were made using repeated measures of ‘analysis of variance (ANOVA)’ and ‘analysis of covariance (ANCOVA)’, respectively. Treatment, visit, and study site were controlled by including them as fixed factors in the analysis. Post-hoc analysis was conducted using Bonferroni’s multiple comparison tests. The results were considered statistically significant at *p* < 0.05.

## Results

### Baseline characteristics

The subjects were randomized and equally distributed among the CL19183 and placebo groups. Their baseline characteristics are summarized in Table 1. A total of 120 subjects were recruited from three study sites: 50 subjects (26-M and 24-F) from Aditya Multispeciality Hospital, 30 subjects (19-M and 11-F) from Samishta Hospital & Research Institute, and 40 subjects (24-M and 16-F) from NUHA Hospitals. Overall, the active group receiving CL19183 (450 mg/day) and placebo were not statistically different at baseline for any of the characteristics assessed. The study scheme, including randomization, follow-up, and efficacy analyses, is depicted in the CONSORT diagram (Fig. 1). During the study, comparisons between the daily average (±SD) of dietary carbohydrates (placebo: 304.77 ± 35.81 g, CL19183: 306.11 ± 34.90 g, *p* = 0.8413), protein (placebo: 58.42 ± 7.84 g, CL19183: 57.96 ± 6.66 g; *p* = 0.7353) and fat (placebo: 52.97 ± 10.99 g; CL19183: 55.28 ± 13.01 g; *p* = 0.3126) and calorie (placebo: 1994.78 ± 198.76 kcal, CL19183: 2018.61 ± 241.16 kcal; *p* = 0.5702) consumptions were not statistically different.

**Table 1:** Baseline characteristics of the placebo and CL19183-supplemented participants

Characteristics	Placebo (*n* = 60)	CL19183 (*n* = 60)	*P*
Gender (M + F)	36 + 24	33 + 27	-
Age (years)	35.7 ± 8.6	35.3 ± 9.1	0.8053
Body weight (kg)	75.7 ± 9.6	73.9 ± 10.0	0.3307
Height (m)	1.6 ± 0.1	1.6 ± 0.1	0.5108
BMI (kg/m^2^)	27.8 ± 1.5	27.5± 1.5	0.3277

Values present as mean ± standard deviation (SD). A *p* value < 0.05 (unpaired t-test) was considered significant. BMI: Body mass index.

### CL19183 supplementation reduced BW and BMI

Sixteen weeks of CL19183 supplementation exhibited mean reductions of 5.75 and 5.70% (*p* < 0.0001) in BW and BMI, respectively, from baseline; in contrast, the placebo group showed only 1.27 and 1.30% reductions (*p* < 0.0001) in BW and BMI, respectively (Table 2). In the CL19183 group, comparison between the changes (from baseline) in BW (0.64 ± 0.54 kg vs. placebo: 0.22 ± 0.25 kg; *p* < 0.0001) and BMI (0.88 ± 0.36 vs. placebo: 0.21 ± 0.36 kg/m^2^; *p* < 0.0001) was significant, as early as 2 and 8 weeks of supplementation, respectively (Table 2).

**Table 2:** Changes in body weight and BMI

Interventions	Evaluation	Net measure	Change from baseline	*P* value (vs. baseline)	*P* value (vs. placebo)	*P* value (between changes from baseline)	95% CI (vs. placebo)
Body weight (kg)							
Placebo	Baseline	75.70 ± 9.62	-	-	-	-	-
	Week 2	75.47 ± 9.61	-0.22 ± 0.25	<0.0001	-	-	-
	Week 4	75.35 ± 9.59	-0.35 ± 0.35	<0.0001	-	-	-
	Week 8	75.11 ± 9.72	-0.58 ± 0.94	<0.0001	-	-	-
	Week 12	74.95 ± 9.78	-0.75 ± 1.01	<0.0001	-	-	-
	Week 16	74.73 ± 9.89	-0.96 ± 1.18	<0.0001	-	-	-
CL19183-450 mg	Baseline	73.95 ± 9.96	-	-	0.3330	-	-1.79, 5.29
	Week 2	73.31 ± 9.76	-0.64 ± 0.54	<0.0001	0.2270	<0.0001	-1.34, 5.66
	Week 4	72.52 ± 9.76	-1.43 ± 0.80	<0.0001	0.1142	<0.0001	-0.67, 6.33
	Week 8	71.54 ± 9.77	-2.41 ± 0.96	<0.0001	0.0482	<0.0001	0.05, 7.09
	Week 12	70.76 ± 9.78	-3.19 ± 1.07	<0.0001	0.0213	<0.0001	0.65, 7.73
	Week 16	69.70 ± 9.83	-4.25 ± 1.35	<0.0001	0.0063	<0.0001	1.47, 8.59
BMI (kg/m^2^)							
Placebo	Baseline	27.79 ± 1.51	-	-	-	-	-
	Week 8	27.58 ± 1.53	-0.21 ± 0.36	<0.0001	-	-	-
	Week 16	27.43 ± 1.62	-0.36 ± 0.46	<0.0001	-	-	-
CL19183 450 mg	Baseline	27.52 ± 1.52	-	-	0.3206	-	-0.30, 0.84
	Week 8	26.64 ± 1.59	-0.88 ± 0.36	<0.0001	0.0011	<0.0001	0.36, 1.52
	Week 16	25.94 ± 1.67	-1.57 ± 0.53	<0.0001	<0.0001	<0.0001	0.87, 2.11

Data present as mean ± SD. Placebo (*n* = 60), CL19183-450 mg (*n* = 60). A *p* value < 0.05 indicates statistical significance; comparison vs. baseline (using ANCOVA model), comparisons vs. placebo, and between changes from baseline (vs. placebo) were analyzed using ANOVA model. BMI: Body mass index.

### CL19183 improved BC and anthropometric parameters

Changes in the participants’ total body fat, LBM, fat percentage, and waist and hip circumferences are summarized in Table 3. At the end of the trial, the CL19183-supplemented group reduced body fat by 4.28 ± 1.56 kg (*p* < 0.0001 vs. baseline), whereas the placebo group reduced only 0.85 ± 1.06 kg (*p* < 0.0001 vs. baseline) (Table 3). CL19183 supplementation resulted in 10.72% (*p* = 0.0074) and 15.03% (*p* = 0.0003) reductions in body fat in weeks 8 and 16, respectively, compared to placebo. Similarly, at the completion of the study, the percent body fat in the CL19183 group was also significantly reduced (41.08 ± 7.44% vs. placebo: 44.95 ± 8.48%; *p* = 0.0095) (Table 3). In the CL19183 group, significant (*p* < 0.0001) reductions in the fat percentage were observed in weeks 8 and 16 of supplementation as compared to baseline (Table 3).

**Table 3:** Body composition and anthropometric measures

Interventions	Evaluation	Net measures	Change from baseline	*P* value (vs. baseline)	*P* value (vs. placebo)	*P* value (between changes from baseline)	95% CI (vs. placebo)
**Total body fat (Kg)**							
Placebo	Baseline	34.58 ± 7.92	-	-	-	-	-
	Week 8	34.24 ± 7.84	-0.34 ± 0.69	0.0076	-	-	-
	Week 16	33.73 ± 8.05	-0.85 ± 1.06	<0.0001	-	-	-
CL19183-450 mg	Baseline	32.93 ± 6.81	-	-	0.2258	-	-1.02, 4.32
	Week 8	30.57 ± 6.90	-2.37 ± 1.14	<0.0001	0.0074	<0.0001	1.00, 6.34
	Week 16	28.66 ± 6.98	-4.28 ± 1.56	<0.0001	0.0003	<0.0001	2.35, 7.79
**Lean body lean (Kg)**							
Placebo	Baseline	38.51 ± 7.35	-	-	-	-	-
	Week 8	38.38 ± 7.47	-0.13 ± 0.59	0.0336	-	-	-
	Week 16	38.37 ± 7.53	-0.14 ± 0.73	0.0514	-	-	-
CL19183-450 mg	Baseline	38.49 ± 7.26	-	-	0.9884	-	-2.62, 2.66
	Week 8	38.60 ± 7.27	0.11 ± 0.29	0.0741	0.8706	0.0061	-2.44, 2.88
	Week 16	38.66 ± 7.26	0.17 ± 0.32	0.0235	0.8294	0.0034	-2.38, 2.96
**Fat percentage (%)**							
Placebo	Baseline	45.59 ± 8.31	-	-	-	-	-
	Week 8	45.44 ± 8.27	-0.15 ± 0.68	0.1697	-	-	-
	Week 16	44.95 ± 8.48	-0.64 ± 1.10	<0.0001	-	-	-
CL19183-450 mg	Baseline	44.55 ± 6.89	-	-	0.4631	-	-1.72, 3.78
	Week 8	42.69 ± 7.20	-1.86 ± 0.99	<0.0001	0.0566	<0.0001	-0.05, 5.55
	Week 16	41.08 ± 7.44	-3.47 ± 1.39	<0.0001	0.0095	<0.0001	0.99, 6.75
**Waist circumference (cm)**							
Placebo	Baseline	97.79 ± 8.27	-	-	-	-	-
	Week 2	97.49 ± 8.33	-0.31 ± 0.51	0.0006	-	-	-
	Week 4	96.91 ± 8.08	-0.89 ± 1.00	<0.0001	-	-	-
	Week 8	96.52 ± 8.26	-1.27 ± 1.32	<0.0001	-	-	-
	Week 12	96.06 ± 8.40	-1.74 ± 1.49	<0.0001	-	-	-
	Week 16	95.70 ± 8.47	-2.10 ± 1.73	<0.0001	-	-	-
CL19183-450 mg	Baseline	96.35 ± 7.89	-	-	0.3294	-	-1.48, 4.36
	Week 2	95.85 ± 7.99	-0.50 ± 0.91	<0.0001	0.2715	0.1164	-1.31, 4.59
	Week 4	94.87 ± 8.06	-1.47 ± 1.40	<0.0001	0.1677	0.0076	-0.88, 4.96
	Week 8	93.59 ± 8.37	-2.76 ± 1.97	<0.0001	0.0533	<0.0001	-0.07, 5.94
	Week 12	92.73 ± 8.46	-3.61 ± 2.23	<0.0001	0.0311	<0.0001	0.28, 6.38
	Week 16	92.02 ± 8.59	-4.32 ± 2.61	<0.0001	0.0186	<0.0001	0.60, 6.76
**Hip circumference (cm)**							
Placebo	Baseline	104.46 ± 7.68	-	-	-	-	
	Week 2	104.27 ± 7.58	-0.19 ± 0.93	0.0763	-	-	
	Week 4	103.94 ± 7.40	-0.52 ± 1.20	0.0019	-	-	
	Week 8	103.69 ± 7.48	-0.77 ± 1.40	0.0005	-	-	
	Week 12	103.30 ± 7.53	-1.16 ± 1.55	<0.0001	-	-	
	Week 16	103.15 ± 7.49	-1.31 ± 1.60	<0.0001	-	-	
CL19183-450 mg	Baseline	102.94 ± 7.71	-	-	0.2923	-	-1.26, 4.30
	Week 2	102.68 ± 7.69	-0.26 ± 0.68	0.0065	0.2647	0.6126	-1.17, 4.35
	Week 4	101.63 ± 7.15	-1.31 ± 1.52	<0.0001	0.0906	0.0028	-0.32, 4.94
	Week 8	100.79 ± 7.42	-2.15 ± 2.20	<0.0001	0.0359	<0.0001	0.21, 5.59
	Week 12	100.24 ± 7.45	-2.70 ± 2.45	<0.0001	0.0271	<0.0001	0.35, 5.77
	Week 16	99.78 ± 7.49	-3.16 ± 2.80	<0.0001	0.0149	<0.0001	0.66, 6.08

Data present as mean ± SD. Placebo (*n* = 60), CL19183-450 mg (*n* = 60). A *p* value < 0.05 indicates statistical significance; comparison vs. baseline (using ANCOVA model), comparisons vs. placebo, and between changes from baseline (vs. placebo) were analyzed using ANOVA model.

Post-trial intergroup comparisons of the net LBM were not significant. However, intergroup comparisons between the mean changes (from baseline) were significant in the 8th week (*p* = 0.0061) and at the end of the study (*p* = 0.0034). At the end of the trial, the LBM was increased by 0.17 ± 0.32 kg (*p* = 0.0235), and in placebo, the LBM was reduced by 0.14 ± 0.73 kg (*p* = 0.0514) from baseline (Table 3).

Furthermore, at the end of the trial period, CL19183 supplementation resulted in significant reductions in waist and hip circumferences, as compared to baseline (*p* < 0.0001) and placebo (waist: *p* = 0.0186; hip: *p* = 0.0149) (Table 3). After 16 weeks of supplementation, the placebo group also showed significant reductions (*p* < 0.0001) in waist and hip circumferences, compared to baseline. In the CL19183 group, the comparison between the changes (from baseline) in waist and hip circumferences was significant (vs. placebo) starting from the 4th week of supplementation (Table 3). However, 16 weeks of CL19183 supplementation did not show significant differences in the waist-to-hip ratio (vs. placebo) (Supplementary Table S2).

### CL19183 supplementation increased RMR and respiratory exchange ratio

CL19183 supplementation significantly increased RMR compared to pre-dose and placebo, starting from baseline through the completion of the study (Table 4). It is interesting to highlight that, at baseline, a single dose (450 mg) of CL19183 significantly (*p* < 0.0001) increased RMR after 60 min (vs. pre-dose) and 120 min (vs. placebo). After 8 and 16 weeks of CL19183 supplementation, the pre-dose RMR values were significantly different from those of placebo. Also, the comparisons between the changes from pre-dose to 180-min post-supplementation (CL19183 vs. placebo) were significant (Table 4). In the 8 and 16 weeks of the study, RER was significantly reduced after CL19183 supplementation (60, 120, and 180 min) compared to baseline and placebo (Table 5).

**Table 4:** Resting metabolic rate

Interventions	Evaluation	RMR (Kcal/day)	Change from baseline	*P* value (vs. baseline)	*P* value (vs. placebo)	*P* value (between changes from baseline)	95% CI (vs. placebo)
**Baseline**							
Placebo	Pre-dose	1330.20 ± 208.39	-	-	-	-	
	60 min	1346.39 ± 230.27	16.19 ± 143.96	0.3223	-	-	
	120 min	1348.57 ± 239.71	18.36 ± 154.66	0.2985	-	-	
	180 min	1347.48 ± 200.68	17.28 ± 165.69	0.3843	-	-	
CL19183-450 mg	Pre-dose	1329.60 ± 133.01	-	-	0.985	-	-62.60, 63.80
	60 min	1406.01 ± 121.39	76.41 ± 117.97	<0.0001	0.0786	0.0139	-6.93, 126.17
	120 min	1511.42 ± 121.60	181.82 ± 131.69	<0.0001	<0.0001	<0.0001	94.13, 231.57
	180 min	1593.95 ± 154.73	264.35 ± 184.47	<0.0001	<0.0001	<0.0001	181.69, 311.25
**Week-8**							
Placebo	Pre-dose	1383.78 ± 117.13	53.58 ± 252.29	0.0002	-	-	-
	60 min	1406.74 ± 133.23	76.54 ± 226.82	<0.0001	-	-	-
	120 min	1408.50 ± 152.73	78.30 ± 244.23	<0.0001	-	-	-
	180 min	1404.66 ± 131.39	74.46 ± 240.39	<0.0001	-	-	-
CL19183-450 mg	Pre-dose	1441.99 ± 96.16	112.39 ± 160.44	<0.0001	0.0037	0.1285	19.47, 96.95
	60 min	1509.05 ± 81.98	179.45 ± 154.17	<0.0001	<0.0001	0.0043	62.32, 142.30
	120 min	1587.02 ± 102.41	257.42 ± 160.61	<0.0001	<0.0001	<0.0001	131.51, 225.53
	180 min	1672.93 ± 86.17	343.33 ± 167.34	<0.0001	<0.0001	<0.0001	228.10, 308.43
**Week-16**							
Placebo	Pre-dose	1388.14 ± 159.63	57.93 ± 261.41	0.0086	-	-	-
	60 min	1408.86 ± 174.79	78.66 ± 250.98	0.0001	-	-	-
	120 min	1439.01 ± 191.22	108.80 ± 272.54	<0.0001	-	-	-
	180 min	1432.65 ± 151.06	102.45 ± 267.32	<0.0001	-	-	-
CL19183-450 mg	Pre-dose	1477.42 ± 179.86	147.82 ± 235.02	<0.0001	0.0049	0.0463	27.80, 150.76
	60 min	1636.26 ± 133.63	306.66 ± 192.13	<0.0001	<0.0001	<0.0001	171.15, 283.65
	120 min	1704.93 ± 137.53	375.33 ± 211.44	<0.0001	<0.0001	<0.0001	205.70, 326.13
	180 min	1789.18 ± 125.84	459.58 ± 199.44	<0.0001	<0.0001	<0.0001	306.27, 406.79

Data present as mean ± SD of RMR before (pre-dose) and post-supplementation (60-, 120, and 180 min). Placebo (*n* = 60), CL19183-450 mg (*n* = 60). A *p* value < 0.05 indicates statistical significance; comparison vs. baseline (using ANCOVA model), comparisons vs. placebo, and between changes from baseline (vs. placebo) were analyzed using the ANOVA model. RMR: resting metabolic rate.

**Table 5:** Respiratory exchange ratio

Interventions	Evaluation	RER	Change from baseline	*P* value (vs. baseline)	*P* value (vs. placebo)	*P* value (between changes from baseline)	95% CI (vs. placebo)
**Baseline**							
Placebo	Pre-dose	0.77 ± 0.05	-	-	-	-	-
	60 min	0.77 ± 0.06	-0.00 ± 0.08	0.9669	-	-	-
	120 min	0.77 ± 0.06	-0.00 ± 0.07	0.9505	-	-	-
	180 min	0.78 ± 0.05	0.01 ± 0.07	0.0401	-	-	-
CL19183-450 mg	Pre-dose	0.76 ± 0.05	-	-	0.4650	-	-0.01, 0.03
	60 min	0.77 ± 0.05	0.01 ± 0.07	0.7972	0.7903	0.4890	-0.02, 0.02
	120 min	0.76 ± 0.04	-0.00 ± 0.07	0.3170	0.4420	0.9712	-0.01, 0.03
	180 min	0.76 ± 0.04	-0.00 ± 0.06	0.2295	0.0162	0.2415	0.00, 0.04
**Week 8**							
Placebo	Pre-dose	0.77 ± 0.06	-0.00 ± 0.08	0.9333	-	-	**-**
	60 min	0.78 ± 0.05	0.01 ± 0.08	0.0039	-	-	-
	120 min	0.77 ± 0.08	0.00 ± 0.08	0.5941	-	-	-
	180 min	0.78 ± 0.05	0.01 ± 0.07	0.0050	-	-	-
CL19183-450 mg	Pre-dose	0.77 ± 0.04	0.00 ± 0.06	0.902	0.9234	0.6485	-0.02, 0.02
	60 min	0.75 ± 0.03	-0.02 ± 0.05	0.0003	<0.0001	0.0144	0.02, 0.04
	120 min	0.75 ± 0.04	-0.02 ± 0.08	0.0147	0.0345	0.2248	-0.00, 0.04
	180 min	0.75 ± 0.04	-0.02 ± 0.06	0.0003	<0.0001	0.0091	0.01, 0.05
**Week 16**							
Placebo	Pre-dose	0.78 ± 0.05	0.01 ± 0.07	0.0323	-	-	-
	60 min	0.79 ± 0.04	0.02 ± 0.06	0.0003	-	-	-
	120 min	0.79 ± 0.05	0.02 ± 0.07	0.0005	-	-	-
	180 min	0.79 ± 0.05	0.02 ± 0.07	<0.0001	-	-	-
CL19183-450 mg	Pre-dose	0.76 ± 0.05	-0.00 ± 0.06	0.5713	0.0569	0.4194	0.00, 0.04
	60 min	0.74 ± 0.04	-0.02 ± 0.06	<0.0001	<0.0001	0.0007	0.04, 0.06
	120 min	0.74 ± 0.05	-0.02 ± 0.08	<0.0001	<0.0001	0.0028	0.03, 0.07
	180 min	0.74 ± 0.04	-0.02 ± 0.07	<0.0001	<0.0001	0.0007	0.03, 0.07

Data are present as mean ± SD of RER before (pre-dose) and post-supplementation (60-, 120, and 180 min). Placebo (*n* = 60), CL19183-450 mg (*n* = 60). A *p* value < 0.05 indicates statistical significance; comparison vs. baseline (using the ANCOVA model), comparisons vs. placebo, and between changes from baseline (vs. placebo) were analyzed using the ANOVA model. RER: respiratory exchange ratio.

### CL19183 improved serum lipid profile and HOMA-IR

Changes in the serum lipid profile of the participants are summarized in Table 6. Overall, the changes in the lipid metabolite levels in the CL19183-supplemented participants were improved as compared to baseline and placebo. At the end of the study, CL19183 supplementation significantly reduced the LDL levels (*p* = 0.0262 vs. baseline; *p* = 0.0340 vs. placebo) and the LDL/HDL ratio (*p* = 0.0022 vs. baseline; *p* = 0.0091 vs. placebo). In placebo, no significant changes in these lipid metabolites were observed (Table 6).

**Table 6:** Serum lipids

Interventions	Evaluation	Mean± SD	Change from baseline	*P* value (vs. baseline)	*P* value (vs. placebo)	*P* value (between changes from baseline)	95% CI (vs. placebo)
**LDL (mg/dL)**							
Placebo	Baseline	110.61 ± 25.69	-	-	-	-	-
	Week 8	111.52 ± 23.80	-0.91 ± 23.25	0.4706	-	-	-
	Week 16	110.67 ± 20.65	0.07 ± 28.65	0.5765	-	-	-
CL19183-450 mg	Baseline	107.17 ± 22.14	-	-	0.4132	-	-5.24, 12.11
	Week 8	105.98 ± 21.98	-1.18 ± 21.68	0.4053	0.1685	0.6035	-2.74, 13.82
	Week 16	103.01 ± 20.61	-4.16 ± 22.89	0.0262	0.0340	0.3580	0.20, 15.12
**HDL (mg/dL)**							
Placebo	Baseline	44.13 ± 8.55	-	-	-	-	-
	Week 8	44.02 ± 7.89	-0.12 ± 6.31	0.644	-	-	-
	Week 16	44.72 ± 6.78	0.58 ± 7.29	0.736	-	-	-
CL19183-450 mg	Baseline	45.13 ± 8.46	-	-	0.4859	-	-2.13, 4.13
	Week 8	45.18 ± 8.73	0.05 ± 8.42	0.704	0.3797	0.902	-1.85, 4.17
	Week 16	46.42 ± 9.56	1.28 ± 8.92	0.0537	0.1857	0.6277	-1.30, 4.70
**LDL/HDL Ratio**							
Placebo	Baseline	2.56 ± 0.67	-	-	-	-	-
	Week 8	2.56 ± 0.55	0.01 ± 0.77	0.2726	-	-	-
	Week 16	2.51 ± 0.54	-0.05 ± 0.77	0.7377	-	-	-
CL19183-450 mg	Baseline	2.40 ± 0.43	-	-	0.1336	-	-0.04, 0.36
	Week 8	2.38 ± 0.45	-0.03 ± 0.55	0.1704	0.0424	0.7672	-0.00, 0.36
	Week 16	2.26 ± 0.49	-0.14 ± 0.63	0.0022	0.0091	0.4611	0.06, 0.44
**VLDL (mg/dL)**							
Placebo	Baseline	27.07 ± 6.10	-	-	-	-	-
	Week 8	27.15 ± 6.19	0.08 ± 5.38	0.6535	-	-	-
	Week 16	27.08 ± 6.33	0.01 ± 6.14	0.7368	-	-	-
CL19183-450 mg	Baseline	26.30 ± 6.58	-	-	0.5013	-	-1.52, 3.06
	Week 8	26.18 ± 5.18	-0.12 ± 6.20	0.6043	0.3476	0.848	-1.09, 3.03
	Week 16	26.18 ± 5.69	-0.12 ± 6.83	0.6256	0.4114	0.9153	-1.28, 3.08
**Triglycerides (mg/dL)**							
Placebo	Baseline	144.30 ± 41.90	-	-	-	-	-
	Week 8	144.22 ± 35.04	-0.08 ± 28.21	0.5909	-	-	-
	Week 16	144.72 ± 33.58	0.42 ± 31.14	0.4199	-	-	-
CL19183-450 mg	Baseline	134.55 ± 31.01	-	-	0.1402	-	-3.58, 23.08
	Week 8	134.42 ± 32.33	-0.13 ± 30.01	0.5467	0.1137	0.9924	-2.39, 21.99
	Week 16	134.37 ± 30.06	-0.18 ± 27.24	0.463	0.075	0.9106	-1.17, 21.87
**Total cholesterol (mg/dL)**							
Placebo	Baseline	182.23 ± 30.45	-	-	-	-	-
	Week 8	182.68 ± 30.27	0.45 ± 21.52	0.6582	-	-	-
	Week 16	185.41 ± 25.52	3.18 ± 29.11	0.1204	-	-	-
CL19183-450 mg	Baseline	177.78 ± 30.99	-	-	0.3927	-	-6.66, 15.56
	Week 8	177.70 ± 30.02	-0.08 ± 26.58	0.7562	0.3302	0.9011	-5.92, 15.88
	Week 16	176.70 ± 29.48	-1.08 ± 28.15	0.4063	0.0592	0.3961	-1.26, 18.68

Data present as mean ± SD. Placebo (*n* = 60), CL19183-450 mg (*n* = 60). A *p* value < 0.05 indicates statistical significance; comparison vs. baseline (using the ANCOVA model), comparisons vs. placebo, and between changes from baseline (vs. placebo) were analyzed using the ANOVA model. LDL: low-density lipoprotein; HDL: high-density lipoprotein; VLDL: very low-density lipoprotein.

In parallel, post-trial, CL19183 supplementation significantly reduced HOMA-IR (*p* = 0.0057 vs. placebo; *p* < 0.0001 vs. baseline). Also, in the 8th week of the study, the reduction of HOMA-IR in the CL19183-supplemented group was significant (*p* = 0.0006 vs. baseline). The changes in HOMA-IR in placebo were not significant during the trial (Table 7).

**Table 7:** Homeostatic model assessment for insulin resistance

Interventions	Evaluation	HOMA-IR	Change from baseline	*P* value (vs. baseline)	*P* value (vs. placebo)	*P* value (between changes from baseline)	95% CI (vs. placebo)
Placebo	Baseline	2.28 ± 0.54	-	-	-	-	-
	Week 8	2.22 ± 0.46	-0.06 ± 0.46	0.2727	-	-	-
	Week 16	2.21 ± 0.40	-0.07 ± 0.42	0.1978	-	-	-
CL19183-450 mg	Baseline	2.24 ± 0.38	-	-	0.5886	-	-0.13, 0.21
	Week 8	2.10 ± 0.28	-0.14 ± 0.42	0.0006	0.0756	0.3247	-0.02, 0.26
	Week 16	2.02 ± 0.34	-0.22 ± 0.49	<0.0001	0.0057	0.0855	0.06, 0.32

Data present as mean ± SD. Placebo (*n* = 60), CL19183-450 mg (*n* = 60). A *p* value < 0.05 indicates statistical significance; comparison vs. baseline (using the ANCOVA model), comparisons vs. placebo, and between changes from baseline (vs. placebo) were analyzed using the ANOVA model. HOMA-IR: homeostatic model assessment for insulin resistance.

### CL19183 modulated serum adiponectin, ghrelin, and GLP-1 levels

**Fig. 2:**
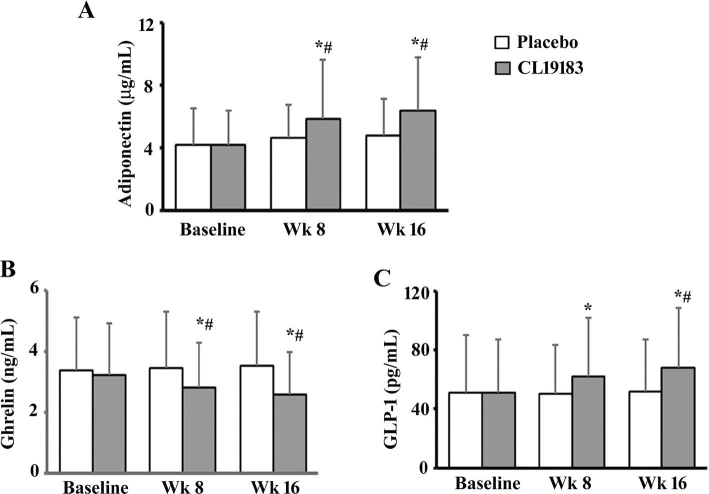
CL19183 modulates serum adiponectin, ghrelin, and total glucagon-like peptide-1 (GLP-1) levels. Each bar presents as mean ± SD of serum adiponectin (A), ghrelin (B), and total GLP-1 (C) levels in the placebo (*n* = 60) and CL19183 (*n* = 60) supplemented participants at baseline, 8th, and 16th weeks of the study. * and # indicate significance (*p* < 0.05) in the ANCOVA model (vs. baseline) and the ANOVA model (vs. placebo), respectively.

Serum adiponectin levels were significantly (*p* < 0.0001) increased in CL19183-supplemented subjects by 40.48 and 51.90% in the 8th and 16th week, respectively, compared to baseline. CL19183 showed 26.33% (*p* = 0.0250 vs. placebo) and 32.09% (*p* = 0.0038 vs. placebo) increases in adiponectin levels in 8 and 16 weeks, respectively. In the placebo group, the changes in adiponectin levels are minimal and not statistically significant compared to baseline (Fig. 2A).

In the CL19183-supplemented group, serum ghrelin levels were significantly reduced in 8 weeks (*p* = 0.0156 vs. baseline; *p* = 0.0314 vs. placebo) and 16 weeks (*p* = 0.0001 vs. baseline; *p* = 0.0009 vs. placebo). The ghrelin levels did not significantly change in the placebo group (Fig. 2B).

CL19183 supplementation significantly increased serum GLP-1 levels by 20.69% (*p* = 0.0009) in 8 weeks and 33.05% (*p* < 0.0001) in 16 weeks from baseline. Post-trial, the increased GLP-1 level was significant (*p* = 0.0138) as compared to placebo. The changes in the placebo group were not significant, compared to baseline (Fig. 2C).

### CL19183 supplementation improves participants’ mood state

CL19183-supplemented participants significantly reduced the total mood disturbance scores from 2 weeks till the end of the study, compared to baseline and placebo (Supplementary Table S3). At the completion of the study, in the CL19183 group (vs. baseline), the POMS-SF domain scores of anger, confusion, depression, and vigor were significantly reduced. In placebo, no domain scores were significantly changed at the end of the study (Supplementary Table S3).

### Safety measures, adverse events, and dropouts

The vital signs, hematology, complete blood biochemistry, and urinalysis parameters were within the normal ranges (Supplementary Table S4).

No serious adverse events were observed during the study. Minor events reported by the participants include runny nose, headache, nausea, and cough (Supplementary Table S5).

Three participants from CL19183 and five participants from the placebo group did not continue the study after the 4th visit. Fifty-seven subjects in the CL19183 group and 55 subjects in the placebo group completed the study.

## Discussion

Dietary supplements containing thermogenic ingredients are increasingly popular among overweight and fitness-conscious individuals to enhance their RMR and facilitate fat loss. Many investigations demonstrate that commercially available thermogenic ingredients boost RMR in obese or overweight subjects (26, 27). However, its regular consumption may negatively affect BC (28, 29). The loss of LBM could compromise the potential health benefits of supplement-induced weight loss (28, 30), which could increase the risk of sarcopenia (31, 32) and deleterious metabolic consequences (33, 34). Hence, it is important to achieve weight loss with a high-fat-to-lean mass loss ratio, which is essential for both the short-term and long-term efficacy of metabolic health and BW regulation (28).

One of the major highlights of the present study is the significant reduction of baseline BW in the active cohort. A 5–10% weight loss has been considered by the United States National Institute of Health–National Heart, Lung, and Blood Institute (NHLBI) to be clinically significant. Moreover, a more than 5% reduction in BW is associated with improved metabolic and cardiovascular health (35). In the present investigation, 16 consecutive weeks of CL19183 supplementation yielded an average of 5.75% BW reduction from the baseline, establishing a potential clinical efficacy of this novel phytoceutical composition.

Another important observation from this study is that the CL19183-supplemented participants exhibited an increased RMR (pre- vs. post-dose) at baseline and after 16 weeks of supplementation. In addition, it is worth noting that post-trial, the pre-dose RMR was significantly increased compared to the pre-dose data at baseline. These observations affirm an acute and long-term effect of CL19183 supplementation in enhancing the REE in the participants. The present observation corroborates with the earlier acute cross-over study (12). It further validates the earlier findings that demonstrated the enhanced thermogenic efficacy of CL19183 *in vitro* and *in vivo* (11). Body size and BC are the determinants of RMR (36); individuals with a reduced RMR pose a higher risk of significant weight gain than those with an increased RMR (37). A series of underlying mechanisms, such as mitochondrial dysfunction, mitochondrial degeneration, and down-regulation of genes responsible for metabolism, may involve decreasing RMR in overweight or obese individuals (38, 39).

Furthermore, the calorimetric evaluation revealed a significant reduction in the estimated RER in the CL19183-supplemented participants. A lower RER indicates increased fat oxidation, thus a greater reduction in adiposity (40). In the present trial, greater reduction of fat mass and improved BC, including anthropometric measures in the CL19183-supplemented participants, might be an effect of increased fat burning due to a thermogenic action of the supplement as explained in earlier investigations (11, 12). Our observations also demonstrate an effect of CL19183 in reducing visceral or central obesity in the participants, suggesting a potential thermogenic role of herbal supplementation. WC and waist-hip ratio (WHR) are used as surrogate markers of visceral adiposity (41, 42). Visceral obesity is a significant risk factor for cardiovascular morbidity and mortality; therefore, increased WC is considered one of the vital diagnostic criteria for metabolic dysfunction (43). There was no difference in net LBM between the groups; however, significant increases between the changes from baseline in the CL19183-supplemented subjects (vs. placebo) suggest that the botanical ingredient has no deleterious effects on muscle mass in overall BW reduction.

In the present investigation, CL19183 supplementation exhibited diverse metabolic benefits, including improved serum lipid profile and insulin sensitization. This phytoceutical significantly reduced serum LDL levels and LDL/HDL ratio in concurrence with the gradual reduction of adiposity and reduced HOMA-IR over the trial period. In general, hyperlipidemia is associated with overweight and obesity, which is considered a potential risk factor for cardiovascular disorders (CVDs), including atherosclerosis (44, 45). The reduced level of circulatory LDL and LDL/HDL ratio indicates an improved status of fat metabolism and a favorable lipoprotein profile. Also, reduced HOMA-IR suggests a possible improvement in the participants’ insulin sensitization and pancreatic beta cell function. Increased HOMA-IR is closely associated with central obesity and a measure of insulin resistance (46–48). The benefit of CL19183 supplementation in metabolic function is further supported by the observation of increased serum adiponectin and reduced ghrelin levels in the participants. Adiponectin, the white adipose tissue-derived endocrine factor, is negatively associated with body fat mass and insulin sensitivity (49). Adiponectin enhances energy production through fatty acid oxidation via 5’ AMP-activated protein kinase (AMPK) activation (50, 51) and has been shown to upregulate the genes responsible for browning fat in knock-out animal models (52). Ghrelin, a peptide hormone, promotes triglyceride uptake by fat cells, increases adiposity, and reduces insulin sensitivity (53). The role of GLP-1 agonists with a strategy of its receptor (GLP-1R) activation or sustained levels of increased GLP-1 has been established as a potential therapeutic strategy for weight management and obesity-associated type-2 diabetes (54–56). GLP-1 has been shown to enhance thermogenesis and fat browning via the UCP-1 pathway and hypothalamic AMPK activation (57) and β3-adrenergic stimulation (58), and its receptor activation has been proposed as a potential target for the treatment of adiposity via thermogenesis (59). Earlier, Kundimi et al. demonstrated that CL19183 enhanced thermogenesis by activating the FGF-21/β3-AR/UCP-1 pathway (11). The increase in GLP-1 levels and the improvements in thermogenesis and fat oxidation observed in the current study suggest a potential role for CL19183 in modulating thermogenesis and fat oxidation via GLP-1. Overall, CL19183 appears to be a multi-mechanistic thermogenic botanical ingredient that combines β3-AR activation with FGF-21, GLP-1, and AMPK pathways, providing a potential advantage over conventional single-pathway thermogenic agents in terms of robust safety, sustained efficacy, and metabolic gain in men and women.

## Conclusion

CL19183 (Theolim™) is a well-tolerated, novel thermogenic supplement that safely promotes weight management by enhancing energy expenditure and fat oxidation without compromising LBM. Unlike conventional thermogenic agents, CL19183 appears to modulate multiple metabolic pathways, including AMPK activation, β3-AR stimulation, and GLP-1 upregulation, supporting its efficacy in reducing adiposity and improving lipid profiles and insulin sensitivity and suggesting potential benefits for obesity-related conditions such as type 2 diabetes and cardiovascular disease.

## Supplementary Material


