# Supplementary material for: A thermogenic botanical composition containing Citrus aurantifolia fruit rind and Theobroma cacao seed extracts improves body composition in overweight adults: a clinical investigation
Source: Food Nutr Res. 2025 Jun 24;69:10.29219/fnr.v69.12159. doi: 10.29219/fnr.v69.12159 (PMC12255159; doi:10.29219/fnr.v69.12159)
Supplement: Supplementary file 1 [file FNR-69-12159-s1.docx]

**Supplementary Table S1: Inclusion-exclusion criteria**

| **Inclusion criteria** |
| --- |
| - Healthy male and female subjects between 25-55 years with body mass index of 25 - 29.9 kg/m^2^. |
| - Subjects with a sedentary lifestyle, no regular athletic or sports activities. - Subjects willing to participate in walking exercise (5 days a week, 30 min per day) over the study duration. |
| - Normal thyroid hormone profile and electrocardiogram (ECG) |
| - Healthy as per health history and routine clinical investigations during screening. |
| - Subjects willing not to consume coffee, tea, cola, energy drinks, chocolate before 24 hours of randomization and evaluation days of the study |
| - Subjects agreed to sign the written informed consent and maintain daily diary and diet tracker. |
| - A negative pregnancy test during recruitment, no lactating mothers, and willing to use medically acceptable contraception for female participants |
| **Exclusion criteria** |
| - Smoking and/or alcohol consumption habits |
| - Consumption of nutritional supplements (e.g., creatine, protein drinks, amino acids, or vitamins) or ergogenic aids within the past 30 days and during the study. |
| - Subjects who participated in another clinical trial within 30 days prior to screening. |
| - Medical condition including metabolic disorders (obesity, hyperlipidemia, diabetes, hypertension, atherosclerosis), arthritis, cancer, or taking medications including, antidepressants, beta-blockers, hormones therapies, etc. |
| - Subjects underwent treatment for COVID-19 or tested positive COVID-19 or HIV during screening |

**Supplementary Table S2: Waist to Hip Ratio**

| Interventions | Evaluation | Waist to Hip Ratio  Mean ± SD | *p*-value  (vs. baseline) | *p*-value  (vs. placebo) | 95% CI  (vs. placebo) |
| --- | --- | --- | --- | --- | --- |
| Placebo | Baseline | 0.94 ± 0.07 | - | - | - |
|  | Week 2 | 0.94 ± 0.07 | 0.1421 | - | - |
|  | Week 4 | 0.93 ± 0.07 | 0.0128 | - | - |
|  | Week 8 | 0.93 ± 0.07 | 0.0051 | - | - |
|  | Week 12 | 0.93 ± 0.07 | 0.0044 | - | - |
|  | Week 16 | 0.93 ± 0.07 | 0.0004 | - | - |
| CL19183-450 mg | Baseline | 0.94 ± 0.10 | - | 0.8779 | -0.03, 0.03 |
|  | Week 2 | 0.94 ± 0.10 | 0.0085 | 0.9329 | -0.02, 0.03 |
|  | Week 4 | 0.94 ± 0.10 | 0.0731 | 0.8227 | -0.02, 0.03 |
|  | Week 8 | 0.93 ± 0.10 | <0.0001 | 0.9953 | -0.02, 0.023 |
|  | Week 12 | 0.93 ± 0.11 | <0.0001 | 0.8847 | -0.02, 0.03 |
|  | Week 16 | 0.93 ± 0.11 | <0.0001 | 0.8554 | -0.02, 0.03 |

Data present mean ± SD. Placebo (n = 60), CL19183-450 mg (n = 60). Comparisons vs. baseline and vs. placebo were analyzed using the ANCOVA and ANOVA model, respectively; a *p* value < 0.05 is considered statistically significant.

**Supplementary Table S3: Profile of Mood States-Short Form (POMS-SF) scores**

| Interventions | Evaluation | | mean± SD | | | Change from baseline  mean± SD | | *p* value  (vs. baseline) | *p* value  (vs. placebo) | *p* value  (Between changes from baseline) | 95% CI  (vs. placebo) |
| --- | --- | --- | --- | --- | --- | --- | --- | --- | --- | --- | --- |
| **Anger** | | |  |  |  |  |  |  |  |  |  |
| Placebo | Baseline | | 7.60 ± 3.22 | | | - | | - | - | - | - |
|  | Week 2 | | 7.98 ± 3.15 | | | 0.38 ± 3.09 | | 0.3329 | - | - | - |
|  | Week 4 | | 7.65 ± 3.04 | | | 0.05 ± 3.85 | | 0.8428 | - | - | - |
|  | Week 8 | | 7.70 ±3 .10 | | | 0.10 ± 3.87 | | 0.8261 | - | - | - |
|  | Week 12 | | 7.77 ± 3.58 | | | 0.17 ± 3.40 | | 0.9284 | - | - | - |
|  | Week 16 | | 7.22 ± 2.60 | | | -0.38 ± 2.97 | | 0.0505 | - | - | - |
| CL19183  450 mg | Baseline | | 8.08 ± 3.19 | | | - | | - | 0.4086 | - | -0.68, 1.64 |
|  | Week 2 | | 7.82 ± 2.55 | | | -0.27 ± 2.10 | | 0.5711 | 0.7493 | 0.1709 | -0.88, 1.20 |
|  | Week 4 | | 7.75 ± 2.91 | | | -0.33 ± 1.48 | | 0.4872 | 0.8530 | 0.4615 | -0.98, 1.18 |
|  | Week 8 | | 7.73 ± 2.15 | | | -0.35 ± 3.01 | | 0.5837 | 0.9455 | 0.4661 | -0.93, 0.99 |
|  | Week 12 | | 7.75 ± 2.25 | | | -0.33 ± 3.06 | | 0.5693 | 0.9757 | 0.3916 | -1.06, 1.10 |
|  | Week 16 | | 7.22 ± 1.99 | | | -0.87 ± 3.61 | | 0.0186 | 1.0000 | 0.4163 | -0.84, 0.84 |
| **Confusion** | | | |  |  |  |  |  |  |  |  |
| Placebo | Baseline | | 3.73 ± 1.93 | | | - | | - | - | - | - |
|  | Week 2 | | 4.02 ± 2.11 | | | 0.28 ± 1.61 | | 0.0943 | - | - | - |
|  | Week 4 | | 4.22 ± 2.03 | | | 0.48 ± 2.76 | | 0.0295 | - | - | - |
|  | Week 8 | | 4.13 ± 2.08 | | | 0.40 ± 2.78 | | 0.0580 | - | - | - |
|  | Week 12 | | 3.47 ± 2.00 | | | -0.27 ± 2.21 | | 0.2183 | - | - | - |
|  | Week 16 | | 3.65 ± 1.91 | | | -0.08 ± 1.91 | | 0.7504 | - | - | - |
| CL19183  450 mg | Baseline | | 3.68 ± 1.90 | | | - | | - | 0.8845 | - | -0.64, 0.74 |
|  | Week 2 | | 3.58 ± 1.73 | | | -0.10 ± 1.20 | | 0.5355 | 0.2145 | 0.1433 | -0.26, 1.14 |
|  | Week 4 | | 3.78 ± 1.85 | | | 0.10 ± 0.73 | | 0.7014 | 0.2262 | 0.3029 | -0.26, 1.14 |
|  | Week 8 | | 3.27 ± 1.38 | | | -0.42 ± 1.37 | | 0.0489 | 0.0085 | 0.0442 | 0.22, 1.50 |
|  | Week 12 | | 3.55 ± 1.41 | | | -0.13 ± 1.64 | | 0.4640 | 0.7899 | 0.7091 | -0.55, 0.71 |
|  | Week 16 | | 3.13 ± 1.42 | | | -0.55 ± 2.21 | | 0.0066 | 0.0919 | 0.2196 | -0.09, 1.13 |
| **Depression** | |  |  |  |  |  |  |  |  |  |  |
| Placebo | Baseline | | | | 5.73 ± 2.57 | | - | - | - | - | - |
|  | Week 2 | | | | 5.92 ± 2.31 | | 0.18 ± 1.77 | 0.3301 | - | - | - |
|  | Week 4 | | | | 6.00 ± 2.25 | | 0.27 ± 2.92 | 0.3918 | - | - | - |
|  | Week 8 | | | | 5.97 ± 2.21 | | 0.23 ± 2.91 | 0.4726 | - | - | - |
|  | Week 12 | | | | 6.12 ± 2.31 | | 0.38 ±2.69 | 0.1763 | - | - | - |
|  | Week 16 | | | | 5.52 ± 2.83 | | -0.22 ±2.73 | 0.2895 | - | - | - |
| CL19183  450 mg | Baseline | | | | 5.90 ± 2.32 | | - | - | 0.7013 | - | -0.72, 1.06 |
|  | Week 2 | | | | 5.60 ± 2.48 | | -0.30 ±0.91 | 0.0998 | 0.4630 | 0.0604 | -0.55, 1.19 |
|  | Week 4 | | | | 5.73 ± 2.48 | | -0.17 ±1.54 | 0.6333 | 0.5377 | 0.3117 | -0.59, 1.13 |
|  | Week 8 | | | | 5.62 ± 1.68 | | -0.28 ±2.12 | 0.3530 | 0.3162 | 0.2726 | -0.36, 1.06 |
|  | Week 12 | | | | 5.53 ± 1.76 | | -0.37 ±2.25 | 0.1995 | 0.1061 | 0.1042 | -0.15, 1.33 |
|  | Week 16 | | | | 5.27 ± 1.41 | | -0.63 ±2.37 | 0.0271 | 0.5193 | 0.3797 | -0.56, 1.06 |
| **Fatigue** | | | | | | | |  |  |  |  |
| Placebo | Baseline | | | | 6.93 ± 2.69 | | - | - | - | - | - |
|  | Week 2 | | | | 7.27 ± 2.17 | | 0.33 ± 1.96 | 0.1169 | - | - | - |
|  | Week 4 | | | | 7.17 ± 2.07 | | 0.23 ± 2.76 | 0.2770 | - | - | - |
|  | Week 8 | | | | 7.08 ± 2.06 | | 0.15 ± 2.60 | 0.4927 | - | - | - |
|  | Week 12 | | | | 6.87 ± 2.18 | | -0.07 ± 3.01 | 0.7974 | - | - | - |
|  | Week 16 | | | | 6.82 ± 2.74 | | -0.12 ± 3.83 | 0.7089 | - | - | - |
| CL19183  450 mg | Baseline | | | | 6.92 ± 2.66 | | - | - | 0.9730 | - | -0.96, 0.98 |
|  | Week 2 | | | | 6.53 ± 2.74 | | -0.38 ± 1.68 | 0.0722 | 0.1084 | 0.0331 | -0.15, 1.63 |
|  | Week 4 | | | | 6.98 ± 2.53 | | 0.07 ± 0.48 | 0.7705 | 0.6654 | 0.6445 | -0.65, 1.03 |
|  | Week 8 | | | | 6.77 ± 2.48 | | -0.15 ± 1.39 | 0.4927 | 0.4490 | 0.4319 | -0.51, 1.13 |
|  | Week 12 | | | | 6.65 ± 2.27 | | -0.27 ± 1.49 | 0.2649 | 0.5967 | 0.6390 | -0.58, 1.02 |
|  | Week 16 | | | | 6.75 ± 2.49 | | -0.17 ± 1.37 | 0.5685 | 0.8893 | 0.9215 | -0.88, 1.02 |
| **Tension** | | | | | | | | | | | |
| Placebo | Baseline | | | | 6.72 ± 3.18 | | - | - | - | - | - |
|  | Week 2 | | | | 7.12 ± 3.17 | | 0.40 ± 2.38 | 0.0900 | - | - | - |
|  | Week 4 | | | | 7.25 ± 3.07 | | 0.53 ± 3.33 | 0.0529 | - | - | - |
|  | Week 8 | | | | 7.17 ± 3.02 | | 0.45 ± 3.39 | 0.0945 | - | - | - |
|  | Week 12 | | | | 6.87 ± 3.06 | | 0.15 ± 2.86 | 0.4979 | - | - | - |
|  | Week 16 | | | | 6.65 ± 3.27 | | -0.07 ± 3.15 | 0.9121 | - | - | - |
| CL19183  450 mg | Baseline | | | | 6.52 ± 3.32 | | - | - | 0.7352 | - | -0.98, 1.38 |
|  | Week 2 | | | | 6.63 ± 3.14 | | 0.12 ± 1.63 | 0.6969 | 0.4045 | 0.4396 | -0.65, 1.63 |
|  | Week 4 | | | | 6.20 ± 3.17 | | -0.32 ± 1.00 | 0.2308 | 0.0693 | 0.0570 | -0.08, 2.18 |
|  | Week 8 | | | | 6.37 ± 2.80 | | -0.15 ± 1.59 | 0.5130 | 0.1365 | 0.2077 | -0.25, 1.85 |
|  | Week 12 | | | | 6.77 ± 2.94 | | 0.25 ± 1.61 | 0.4139 | 0.8553 | 0.8105 | -0.98, 1.18 |
|  | Week 16 | | | | 6.13 ± 2.93 | | -0.38 ± 1.58 | 0.1533 | 0.3597 | 0.4901 | -0.60, 1.64 |
| **Vigor** | | | | | | | | | | | |
| Placebo | Baseline | | | | 9.87 ± 4.10 | | - | - | - | - | - |
|  | Week 2 | | | | 9.83 ± 3.91 | | -0.03 ±3.67 | 0.9229 | - | - | - |
|  | Week 4 | | | | 10.57 ± 4.48 | | 0.70 ± 6.47 | 0.1129 | - | - | - |
|  | Week 8 | | | | 10.87 ± 3.84 | | 1.00 ± 5.86 | 0.0166 | - | - | - |
|  | Week 12 | | | | 10.28 ± 3.58 | | 0.42 ± 5.12 | 0.2078 | - | - | - |
|  | Week 16 | | | | 10.33 ± 4.70 | | 0.47 ± 4.64 | 0.2229 | - | - | - |
| CL19183  450 mg | Baseline | | | | 9.33 ± 3.74 | | - | - | 0.4537 | - | -0.88, 1.96 |
|  | Week 2 | | | | 10.93 ± 4.57 | | 1.60 ± 2.74 | 0.0002 | 0.1534 | 0.0070 | -0.44, 2.64 |
|  | Week 4 | | | | 12.07 ± 4.43 | | 2.73 ± 3.01 | <0.0001 | 0.0663 | 0.0301 | -0.11, 3.11 |
|  | Week 8 | | | | 12.02 ± 4.07 | | 2.68 ± 4.97 | <0.0001 | 0.1158 | 0.0949 | -0.28, 2.58 |
|  | Week 12 | | | | 12.53 ± 4.25 | | 3.20 ± 5.20 | <0.0001 | 0.0024 | 0.0042 | 0.83, 3.67 |
|  | Week 16 | | | | 11.98 ± 4.08 | | 2.65 ± 5.81 | <0.001 | 0.0396 | 0.0251 | 0.06, 3.24 |
| **Total Mood Disturbance** | | | | | | | | | | | |
| Placebo | Baseline | | | | 20.85 ± 5.87 | | - | - | - | - | - |
|  | Week 2 | | | | 22.47 ± 5.86 | | 1.62 ± 5.28 | 0.0056 | - | - | - |
|  | Week 4 | | | | 21.72 ± 5.55 | | 0.87 ± 5.81 | 0.1575 | - | - | - |
|  | Week 8 | | | | 21.18 ± 5.78 | | 0.33 ± 5.87 | 0.7604 | - | - | - |
|  | Week 12 | | | | 20.80 ± 6.94 | | -0.05 ± 5.84 | 0.7752 | - | - | - |
|  | Week 16 | | | | 19.52 ± 4.55 | | -1.33 ± 5.19 | 0.0045 | - | - | - |
| CL19183  450 mg | Baseline | | | | 21.58 ± 5.47 | | - | - | 0.4643 | - | -1.32, 2.78 |
|  | Week 2 | | | | 19.23 ± 5.48 | | -2.35 ± 3.66 | <0.0001 | 0.0023 | <0.0001 | 1.19, 5.29 |
|  | Week 4 | | | | 18.38 ± 5.64 | | -3.20 ± 2.99 | <0.0001 | 0.0015 | <0.0001 | 1.32, 5.36 |
|  | Week 8 | | | | 17.73 ± 5.15 | | -3.85 ± 4.12 | <0.0001 | 0.0008 | <0.0001 | 1.47, 5.43 |
|  | Week 12 | | | | 17.72 ± 5.29 | | -3.87 ± 4.51 | <0.0001 | 0.0057 | 0.0001 | 0.85, 5.31 |
|  | Week 16 | | | | 16.52 ± 5.42 | | -5.07 ± 4.54 | <0.0001 | 0.0010 | <0.0001 | 1.19, 4.81 |

Data present as mean ± SD. Placebo (n = 60), CL19183-450 mg (n = 60). A *p* value < 0.05 indicates statistical significance; comparison vs. baseline (using ANCOVA model), comparisons vs. placebo and between changes from baseline (vs. placebo) were analyzed using ANOVA model

**Supplementary Table S4: Safety parameters**

|  | **Parameters** | **Groups** | **Screening** | **Week 16** |
| --- | --- | --- | --- | --- |
| Serum biochemistry | Fasting Blood Glucose (mg/dL) | Placebo | 96.60 ±7.47 | 94.87 ± 8.43 |
|  |  | CL19183 | 96.82 ±9.66 | 91.23 ± 5.56 |
|  | Creatinine (mg/dL) | Placebo | 0.90 ± 0.20 | 0.94 ± 0.17 |
|  |  | CL19183 | 0.90 ± 0.17 | 0.90 ± 0.17 |
|  | Blood Urea Nitrogen (mg/dL) | Placebo | 13.80 ± 5.49 | 14.28 ± 5.21 |
|  |  | CL19183 | 14.78 ± 6.92 | 14.69 ± 5.65 |
|  | Uric Acid (mg/dL) | Placebo | 5.47 ± 1.39 | 5.69 ± 1.26 |
|  |  | CL19183 | 5.69 ± 1.58 | 5.27 ± 1.34 |
|  | Sodium (mmol/L) | Placebo | 141.17 ± 4.49 | 142.04 ± 4.00 |
|  |  | CL19183 | 141.37 ± 4.33 | 141.75 ± 4.70 |
|  | Potassium (mmol/L) | Placebo | 4.18 ± 0.38 | 4.27 ± 0.43 |
|  |  | CL19183 | 4.13 ± 0.26 | 4.18 ± 0.33 |
|  | Alanine transaminase (IU/L) | Placebo | 29.85 ± 7.99 | 32.24 ± 8.56 |
|  |  | CL19183 | 30.57 ± 8.85 | 32.65 ± 9.70 |
|  | Aspartate aminotransferase (IU/L) | Placebo | 28.57 ± 9.67 | 29.73 ± 9.05 |
|  |  | CL19183 | 28.88 ± 8.55 | 28.65 ± 7.65 |
|  | Alkaline phosphatase (IU/L) | Placebo | 87.90 ± 20.50 | 92.10 ± 20.81 |
|  |  | CL19183 | 93.05 ± 18.96 | 100.98 ± 22.05 |
|  | Bilirubin (mg/dL) | Placebo | 0.78 ± 0.29 | 0.76 ± 0.28 |
|  |  | CL19183 | 0.81 ± 0.34 | 0.76 ± 0.25 |
|  | Albumin (g/dL) | Placebo | 4.16 ± 0.29 | 4.25 ± 0.29 |
|  |  | CL19183 | 4.19 ± 0.25 | 4.13 ± 0.33 |
|  | Creatinine kinase (mg/dL) | Placebo | 69.69 ± 39.73 | 73.09 ± 43.07 |
|  |  | CL19183 | 70.99 ± 37.66 | 66.28 ± 38.77 |
| Hematology | Hemoglobin (g/dL) | Placebo | 13.47 ± 1.82 | 13.44 ± 1.78 |
|  |  | CL19183 | 13.24 ± 1.93 | 13.12 ± 1.58 |
|  | Platelet count (10^5^ /cu.mm) | Placebo | 3.06 ± 0.63 | 3.23 ± 0.58 |
|  |  | CL19183 | 3.04 ± 0.72 | 3.18 ± 0.69 |
|  | Erythrocyte sedimentation rate (mm/hr) | Placebo | 14.46 ± 4.89 | 13.66 ± 4.51 |
|  |  | CL19183 | 15.45 ± 6.17 | 13.43 ± 5.06 |
|  | RBC count (10^6^/cu.mm) | Placebo | 4.70 ± 0.56 | 4.66 ± 0.44 |
|  |  | CL19183 | 4.70 ± 0.58 | 4.72 ± 0.52 |
|  | WBC (cells/cu.mm) | Placebo | 7568 ± 1706 | 7042 ± 1673 |
|  |  | CL19183 | 7492 ± 1884 | 7432 ± 1790 |
|  | Neutrophil (%) | Placebo | 55.22 ±7.91 | 58.15 ± 5.57 |
|  |  | CL19183 | 55.58 ±6.95 | 57.22 ± 6.80 |
|  | Lymphocytes (%) | Placebo | 35.12 ±7.57 | 32.81 ± 5.45 |
|  |  | CL19183 | 35.25 ±5.66 | 33.75 ± 5.73 |
|  | Eosinophil (%) | Placebo | 3.59 ± 1.86 | 3.32 ± 1.59 |
|  |  | CL19183 | 2.97 ± 1.54 | 3.25 ± 1.52 |
|  | Monocytes (%) | Placebo | 5.62 ± 2.07 | 5.12 ± 2.20 |
|  |  | CL19183 | 5.75 ± 2.40 | 5.33 ± 2.26 |
|  | Basophils (%) | Placebo | 0.46 ± 0.52 | 0.60 ± 0.50 |
|  |  | CL19183 | 0.45 ± 0.52 | 0.45 ± 0.52 |
| Vital signs | Pulse rate (beats/minute) | Placebo | 89.7 ± 8.9 | 85.5 ± 5.2 |
|  |  | CL19183 | 89.6 ± 7.6 | 84.9 ± 5.9 |
|  | Systolic BP (mmHg) | Placebo | 122.2 ± 5.7 | 123.1 ± 4.4 |
|  |  | CL19183 | 122.1 ± 5.6 | 122.8 ± 4.6 |
|  | Diastolic BP (mmHg) | Placebo | 80.7 ± 4.7 | 80.3 ± 3.0 |
|  |  | CL19183 | 79.6 ± 4.9 | 80.1 ± 3.2 |
|  | Respiratory rate (breathes/minute) | Placebo | 16.7 ± 2.0 | 16.3 ± 2.2 |
|  |  | CL19183 | 16.5 ± 2.0 | 16.5 ± 2.1 |
|  | Oral temperature (ºF) | Placebo | 97.5 ± 1.3 | 97.8 ± 1.0 |
|  |  | CL19183 | 97.5 ± 1.4 | 97.8 ± 1.1 |

Data present as mean ± SD. At baseline: Placebo (n = 60), CL19183-450 mg (n = 60); at week 16: Placebo (n = 55), CL19183-450 (n = 57).

**Supplementary Table S5:**

| Adverse events | Placebo (n = 60) | CL19183 (n=60) |
| --- | --- | --- |
| Total number of adverse events | 4 | 4 |
| Total number of subjects with adverse events | 4 | 2 |
| Runny Nose | 1 | 1 |
| Headache | 0 | 1 |
| Nausea | 1 | 1 |
| Cough | 1 | 1 |
| Body pain | 1 | 0 |
